# Medical damage liability risk of medical AI: from the perspective of DeepSeek’s large-scale deployment in Chinese hospitals

**DOI:** 10.3389/fpubh.2025.1726205

**Published:** 2025-11-26

**Authors:** Ye Wang, Zishi Zhou

**Affiliations:** 1 School of Law and Public Administration, Hunan University of Science and Technology, Xiangtan, China; 2 Law School, Hunan University, Changsha, China

**Keywords:** medical AI, DeepSeek, legal risk, medical damage liability, reasonable doctor standards

## Abstract

The field of healthcare is one of the important areas for the application of artificial intelligence (AI). This study introduces the current deployment of the AI model DeepSeek in Chinese hospitals, raises concerns about the ethical and legal aspects of medical AI, and identifies the problem of insufficient regulation by reviewing the current regulatory status of medical AI in China. In the discussion section, this article mainly focuses on three types of medical damage liability risks in medical AI, namely medical product liability, diagnosis and treatment damage liability, and medical ethics liability. In the determination of medical product liability, the ethical attributes and technological characteristics of medical AI determine its auxiliary positioning, but the auxiliary positioning of medical AI has not eliminated the applicable space of medical product liability, and in the judgment of product defects, the “rational algorithm” standard based on the “rational person” standard should be used to identify AI design defects; In the determination of diagnosis and treatment damage liability, medical AI has not changed the existing doctor-patient relationship structure, but the human-machine collaborative diagnosis and treatment model has intensified the difficulty of identifying doctor’s fault, so “reasonable doctor” standards should be adopted, and medical personnel should be given the discretion to reevaluate the negligence of doctors in using AI recommendations. In the case of localizing DeepSeek deployment in hospitals, if misdiagnosis occurs, hospitals and doctors are more likely to bear the diagnosis and treatment damage liability rather than medical product liability. At the same time, the adoption of DeepSeek exacerbates the lack of protection for patients’ right to informed consent, which may lead to medical ethical liability. In addition, this article also discusses the data compliance risks of large-scale deployment of DeepSeek in hospitals.

## 1 Introduction

Medical AI is driving the scientific and intelligent development of modern healthcare systems (1) through various technological changes such as the accuracy of medical diagnosis (2), innovation in diagnosis and treatment models, and foresight in health management. With the continuous development of smart healthcare technology and strong policy support, the scale of China’s smart healthcare market is showing a high-speed growth trend. The “Research Report on the Development Trends and Investment Risks of China’s Smart Healthcare Industry from 2025 to 2030” released by the China Business Industry Research Institute shows that the market size of China’s smart healthcare industry will reach 6.285 billion yuan in 2023, with an average annual compound growth rate of 53.37% from 2019 to 2023. Analysts from China Business Industry Research Institute predict that the size of China’s smart healthcare market will grow to 11.137 billion yuan in 2024 and reach 12 billion yuan in 2025 (3). At the beginning of 2025, the big language model DeepSeek emerged, and its open-source features and low-cost deployment advantages have attracted widespread attention (4). Since the release of DeepSeek R1 on January 20, 2025, in just over 3 months, DeepSeek has created an industry legend. As of March, Quest Mobile data shows that its eponymous app has become the largest AI native application in China with over 190 million monthly active users (5). Major hospitals have deployed DeepSeek one after another, hoping to improve their medical service level with the help of DeepSeek (6). The localization deployment of DeepSeek in hospitals which can be customized and optimized according to the specific needs of the hospital, refers to the installation of DeepSeek large models and their related software and hardware devices on local servers or private clouds in hospitals, enabling hospitals to independently control data and models, improving data security and privacy. However, in the face of such a strong wave of DeepSeek deployment, many people have expressed concerns about ethical and legal risks of AI medical. Scholars are concerned about the user-friendly evaluation, method transparency, and ethical issues of AI in clinical use (7), and some experts have pointed out that the rapid and unregulated adoption of DeepSeek has exceeded China’s overall regulatory supervision and governance framework, resulting in regulatory lag (8). Faced with the concerns and regulatory challenges brought by the large-scale deployment of DeepSeek in hospitals, this study mainly discusses the legal liability risks of medical AI, including medical product liability, diagnosis and treatment damage liability and medical ethic liability of medical AI through analyzing the legal nature of the medical AI, definition of fault liability of infringement and reconstruction of informed consent rules of patients. The data compliance issues of medical AI have also been discussed.

## 2 Materials and methods

### 2.1 Research methodology

This paper mainly conducts speculative research or theoretical research. It mainly involves discussion in a legal and theoretical sense, and the research method is mainly qualitative. This paper did not generate empirical data, but instead constructed its arguments by carefully studying legal texts and related secondary materials, including academic reviews and policy documents. In addition, the data used in the study comes from reliable sources such as publicly news and available legal documents in China.

This paper uses literature research methods and comparative research methods to specifically analyze the proposed research questions. First, this paper mainly adopts the literature research method by collecting, identifying, and organizing documents, to form a scientific understanding of the facts through the study of documents. The range of literature includes papers, reports, legal norms, policy documents and judicial precedents related to the implementation of the medical AI. Through analysis of the literature, we can fully understand the current legal risk and regulatory challenges of medical AI in China. Second, this paper adopts the comparative research method in some parts, and defines the legal liability standard of medical AI in China by comparing the differences in the regulatory of medical AI in some other countries and China.

### 2.2 Materials

The literature collected in this paper is mainly divided into three categories. Table 1 shows the three types of materials in this article: policy documents, legal norms and judicial cases.

**Table 1:** Referenced document types.

Materials type	Documents	Source
Policy documents	Reference Guidelines for Artificial Intelligence; Key Points for the Evaluation of Medical Device Software with Deep Learning Assisted Decision Making. etc.	China Government Network
Legal norms	Civil Code Related Judicial interpretation Departmental rules	China Government Network
Judicial cases	Cases of searching with “medical AI” as a keyword	China Judgment Document Network

### 2.3 Context of the study

#### 2.3.1 Current situation of hospital deployment of DeepSeek

The shortage of medical resources is a long-standing structural problem in China’s healthcare system (9). In the past development stage, medical AI technology mainly relied on medical device carriers such as auxiliary diagnosis and treatment systems and intelligent decision-making platforms to improve diagnostic efficiency and control medical costs during the diagnosis stage (10). It is worth noting that the new generation of AI technology represented by DeepSeek is driving a systematic change in the diagnosis and treatment mode. Through the full process solution of intelligent triage before diagnosis, assisted decision-making during diagnosis, and post diagnosis health management, it further improves the efficiency of diagnosis and treatment. Recently Stanford University has released a comprehensive evaluation of clinical medical AI models, and DeepSeek R1 stands out as the champion among nine cutting-edge models with a winning rate of 66% and a macro average score of 0.75 (11). Since DeepSeek became popular in February 2025, news of major hospitals rushing to deploy DeepSeek locally has been constantly emerging.

Regarding the popularity of DeepSeek, some articles even exaggerate China’s medical AI policies. For example, in March, Gradient Flow published an article titled “DeepSeek in Action: Practical AI Applications Transforming Chinese Healthcare,” which “magnified” the progress of the Chinese healthcare industry in AI applications, and even “accelerated” the medical AI policies. The article mentioned that “China’s National Health Commission (NHC) mandate for AI integration in all tertiary hospitals by 2025” (12), but there is no basis for this statement. On November 6, 2024, in order to implement the decision and deployment of the Party Central Committee and the State Council on the “AI+” action and promote the innovative application of AI in the field of health, the NHC, the State Administration of Traditional Chinese Medicine (SATCM), and the National Bureau of Disease Control jointly formulated and released the “Reference Guidelines for Artificial Intelligence Application Scenarios in the Health Industry” (13), which divided applications into four major fields, 13 categories, and 84 typical application scenarios, actively promoting the innovative development of “AI+” applications in the health industry, and providing scene references and direction guidance for the application of medical AI. The guidelines specify various application scenarios of AI in medical service management, including medical imaging assisted diagnosis, clinical specialized disease intelligent decision-making, and primary general practitioner assisted decision-making. The “2025 Government Work Report” released mentions the need to continue promoting the “AI +” initiative, better combining digital technology with manufacturing and market advantages, and supporting the widespread application of large models. But there is no policy document requiring all tertiary hospitals to integrate AI technology by 2025.

**Figure 1:**
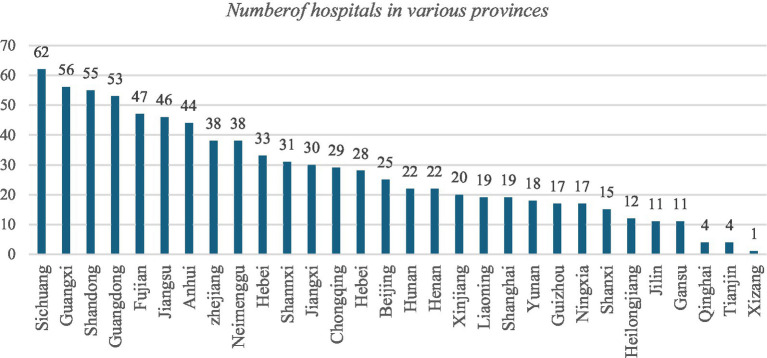
As of May 3, 2025, the number of hospitals in various provinces across the country that have deployed DeepSeek.

As of June 2025, over 830 hospitals have completed the localization deployment of DeepSeek within their facilities, Figure 1 shows the number of hospitals deploying DeepSeek in various provinces of China. More than 50% of the tertiary hospitals (445) have customized different versions of DeepSeek models based on their own situations, and 128 primary hospitals (whose levels are unknown) have deployed DeepSeek (14). The main functions used are medical report interpretation, intelligent guidance, auxiliary diagnosis, case quality control, clinical decision support, case production, intelligent Q&A, and so on. It is also worth noting that the deployment of DeepSeek’s large model in Chinese hospitals has gradually shifted from being “large and comprehensive” to being “specialized and refined.” For example, the neurology department of Huanshan Hospital in Shanghai has integrated DeepSeek for deep customization in vertical fields, while also meeting real clinical needs (15).

#### 2.3.2 Regulatory status of hospital deployment of DeepSeek

In order to standardize the application of medical AI, China has introduced multiple regulations and policies in the past 5 years to manage medical AI (16), such as “Key Points for the Evaluation of Medical Device Software with Deep Learning Assisted Decision Making,” “Ethical Guidelines for New Generation Artificial Intelligence,” and “Guiding Principles for Registration and Review of Artificial Intelligence Medical Devices.” These regulations and guidelines focus on data quality, algorithm generalization ability, clinical use risks, ethical issues, etc., to ensure the safety and reliability of medical AI.

Faced with the large-scale deployment of DeepSeek in hospitals, on March 29, 2025, at the parallel forum-"Medical AI Innovation and Development Forum” of Zhongguancun Annual Meeting, the “Expert Consensus on the Deployment of DeepSeek in Medical Institutions” (17) was officially released to the public. The Consensus was jointly formulated by the Big Data Internet Artificial Intelligence Medical Special Committee of the Beijing Health Law Society and the legal ethics expert group of the Medical Artificial Branch of the Chinese Biomedical Engineering Society. It systematically standardized the deployment process of AI in medical scenarios, emphasizing the improvement of diagnosis and treatment accuracy and the protection of patient privacy and safety through technical standardization and risk control. The consensus proposes that medical institutions need to complete three key assessments before deploying AI systems such as DeepSeek: firstly, Assessment of medical needs adaptability, requiring customized solutions for clinical pain points in different departments; secondly, Data quality and infrastructure assessment, emphasizing the professional cleaning, annotation, and security protection of raw medical record data; thirdly, Legal regulations and ethical risk review, requiring the establishment of a full process compliance mechanism.

From this consensus, it can be seen that in addition to demand analysis and technical requirements, the ethical and legal risks of medical AI are factors that hospitals must consider when deploying DeepSeek. Of course, the ethical issues of medical AI have been widely discussed globally (18). The World Health Organization (WHO) has proposed six principles for the ethical governance of medical AI in its “Ethical Governance of Artificial Intelligence in Healthcare” guidelines (19), including safeguarding human autonomy, enhancing human well-being and protecting safety and public interests, ensuring transparency, interpretability and comprehensibility, developing responsibility and accountability, ensuring inclusiveness and fairness, and promoting responsiveness and sustainability.

In terms of legal and regulatory supervision of medical AI in China, it is still in the exploratory stage. Table 2 shows the current regulations of medical AI and DeepSeek. Regarding the large-scale deployment of DeepSeek in hospitals, the Hunan Provincial Medical Insurance Bureau issued a notice titled “Further Strengthening the Management of Designated Retail Pharmacies for Basic Medical Security” (20) in February 2025, and it stipulates that: Prescription behavior should be effectively and fully communicated with patients or their families for consultation, and the use of AI and other automatic prescription generation is strictly prohibited. This provision is consistent with the provisions of the “Notice on Printing and Distributing the Detailed Rules for the Supervision of Internet Diagnosis and Treatment (Trial)” issued by the General Office of the NHC and the Office of the SATCM in 2022 (21), that is, the prescriptions should be issued by the doctors who receive them, and it is strictly prohibited to use AI to automatically generate prescriptions. Therefore, hospitals should also refer to and pay attention to the localization deployment function of DeepSeek, avoid using DeepSeek for automatic prescription generation, and strictly prohibit pharmacies from accepting AI generated prescriptions. Overall, the medical AI software is in an “auxiliary position” in current legal norms. AI assisted diagnostic technology is an auxiliary diagnosis and clinical decision support system, and cannot be used as the final clinical diagnosis. It is only used as a clinical auxiliary diagnosis and reference, and the final diagnosis must be determined by qualified clinical doctors (22). The convenience and risks brought by medical AI coexist. In the process of medical AI regulation moving from decentralization to systematization, important legal risk prevention is imperative.

**Table 2:** Current regulations of medical AI and DeepSeek deployment in China.

Regulatory category	Medical AI	DeepSeek deployment
Law	Civil Code (2020)	Apply equally
	Basic Healthcare and Health Promotion Law (2019)	Apply equally
Administrative regulations and Departmental regulations	Regulations on the Supervision and Administration of Medical Devices (2021)	Apply equally
	Key Points for the Evaluation of Medical Device Software with Deep Learning Assisted Decision-Making (2019)	Apply equally
	Management Specification for Artificial Intelligence Assisted Diagnosis Technology (2017)	Apply equally
	Ethical Guidelines for New Generation Artificial Intelligence (2021)	Apply equally
	Guiding Principles for Classification and Definition of Artificial Intelligence Medical Software Products (2021)	
	Guiding Principles for Registration and Review of Artificial Intelligence Medical Devices (2022)	Apply equally
Local regulations	Guidelines for Inspection of Quality Management Standards for Artificial Intelligence Medical Device Production in Beijing (2024)	Further Strengthening the Management of Designated Retail Pharmacies for Basic Medical Security of Hunan (2025)
	Implementation Measures of Beijing Municipality on the Supervision of Internet Diagnosis and Treatment (2023)	
Industry Autonomy Rules		Expert Consensus on the Deployment of DeepSeek in Medical Institutions (2025)

### 2.4 Research design

**Figure 2:**
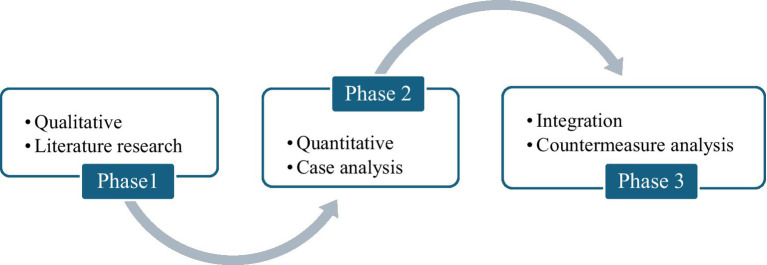
Outline of the multi-staged research work that the current study (phase 1) is a part of.

This article is based on the background of large-scale deployment of DeepSeek in Chinese hospitals, and studies the legal liability risk prevention of medical AI from the perspective of public legal concerns. This is a multi-stage research work (see Figure 2), and this article is the first phase of the research, which is the theoretical research (qualitative research) stage. The second stage will focus on analyzing and quantitatively studying the legal disputes and judicial cases arising from the application of DeepSeek in hospitals. The third stage will integrate the results of the two stages to promote legal or policy progress.

This article is the first phase of theoretical research, and the theoretical framework is based on the theory of the constituent elements of general tort liability. The determination of legal liability for medical AI infringement cannot be separated from the theory of constituent elements of infringement liability. The laws of most countries in the world stipulate four constituent elements of tort liability, namely (1) engaging in civil illegal acts; (2) Other people’s property or personal injury; (3) There is a causal relationship between behavior and the consequences of harm; (4) The perpetrator subjectively has intentional or negligent fault. The illegal behavior (act or omission) and the elements of harm consequences are the result of objective judgment. The application of medical AI makes it more difficult to determine the diagnosis and treatment negligence of medical personnel, and at the same time makes it particularly difficult to determine the legal causal relationship between harm consequences and medical behavior. This difficulty is reflected in the autonomy of AI and the negligence of doctor behavior, which are important factors affecting the subjective fault and causal relationship judgment of medical personnel, and also the key to determining the liability for medical AI infringement.

### 2.5 Data collection and result

#### 2.5.1 Concerns about the large-scale deployment of DeepSeek in hospitals

**Figure 3:**
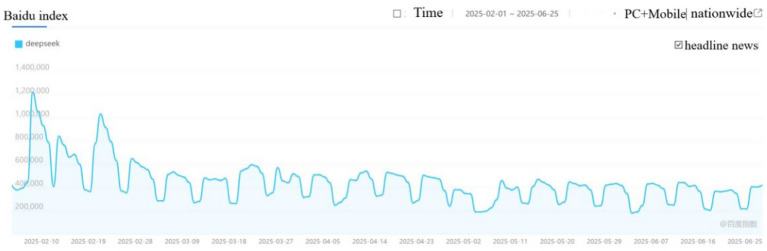
News popularity of DeepSeek from February to June 2025.

With the large-scale deployment of DeepSeek models in major hospitals (the data collection mainly comes from manual sorting of official news released by hospitals), they are used in multiple scenarios such as clinical, scientific research, and administrative management (23). Many patients and researchers have expressed concerns. From February to June2025, in Baidu’s News Popularity Index, searching for the keyword “DeepSeek” can reveal its news popularity (see Figure 3), searching for news content using the keyword DeepSeek medical legal risks, and through manual screening of hot news, public concerns about hospital deployment of DeepSeek can be identified. Some patients wonder: if doctors now use DeepSeek when seeing patients, what is the necessity of doctors? The patient presented the results of DeepSeek to the doctor for discussion, and the doctor responded with DeepSeek, causing the patient to doubt the authority of the diagnostic results. The safety of assisted diagnosis and treatment, the reliability of diagnosis and treatment results, and the acceptability of medical artificial intelligence by the public are the issues of common concern to everyone (24).

With the concerns of the large-scale deployment of DeepSeek in hospitals, although so far, no case of medical liability dispute caused by AI medical software has been retrieved from the publicly available case database in China, the internet is flooded with news of medical AI misdiagnosis causing patient injuries and resulting in infringement lawsuits, such as “AI doctors causing big trouble! A patient in Shanghai was misdiagnosed with pneumonia and narrowly escaped death. Using such news headlines to attract readers and exacerbate patients’ concerns about medical AI. Finally, it was discovered that these were all rumors (25). Moreover, in fake news, detailed descriptions of information such as time, location, parties involved in litigation, courts, and the focus of the case dispute can make people believe it to be true. For example, on March 13, 2025, the first AI medical misdiagnosis lawsuit in China was officially heard at the Haidian District People’s Court in Beijing. The patient’s family claims 3 million yuan, and the hospital, AI research and development company, and attending physician each hold their own opinions. The focus of the dispute in the case is whether there are defects in the algorithm of the “intelligent diagnosis AI system”? Are doctors overly reliant on AI? Are hospitals fulfilling their regulatory responsibilities? (26). Although this case is fake, it reflects the public’s concern about the use of DeepSeek applications in hospitals.

In addition, researchers have conducted extensive research on the potential legal and ethical issues of DeepSeek, which serve as important references for this article. The selection and inclusion process of literature in this article is carried out through two steps. Firstly, a preliminary screening is conducted using keywords. This step involves browsing through the citation information of the detected literature, such as titles and abstracts, to conduct a preliminary screening, removing those that clearly do not meet the requirements, and conducting full-text screening on literature that may meet the criteria. Secondly, when conducting full-text screening, carefully read and evaluate the main content of each literature to determine whether it meets the legal risks and infringement liability issues focused on in this article. For example, for the selection of Chinese literature, a literature search on CNKI was conducted, with a limited time period from January to July 2025, and 145 relevant articles were found using the keywords “medical artificial intelligence” and “law.” Finally, 24 articles related to DeepSeek’s medical legal risk analysis were selected. The researchers concerned about the patient privacy and data security risks, such as medical data leakage, as well as ethical issues related to algorithms, or algorithmic black boxes and biases that may lead to opaque and discriminatory medical decision-making (27). They are also concerned about unclear responsibility delineation between medical AI designers, manufacturers, and users, as well as excessive reliance on AI solutions that may weaken the authority of doctors (28).

#### 2.5.2 The main types of ethical and legal concerns in medical AI

The focus of these issues mainly lies in the practical application of medical AI and its interaction with doctor-patient diagnosis and treatment. At the theoretical research level, due to the high complexity and uncertainty of medical AI technology, the value of “anthropocentrism” highlights issues such as medical ethics, moral ethics, transparency, and interpretability of medical AI. At the same time, it also brings about discussions on legal issues related to medical AI, such as privacy protection, data compliance, protection of patients’ informed consent rights, and determination of tort liability. Table 3 shows the main types of ethical and legal concerns in medical AI.

**Table 3:** Legal and ethical issues of medical AI.

Concerns of medical AI	Legal risk	Privacy protection; data compliance; patients’ informed consent rights; tort liability
	Ethical risk	Autonomy of patients; transparency and explainability; responsibility and accountability; inclusiveness and fairness

In the field of privacy and data protection, the digital management of medical records has brought about data security issues and privacy protection challenges. The rise of remote medical platforms has led to an increasing amount of patient data being collected by proprietary software, which has raised concerns about unknown risks to the safety and privacy information of patients (29). In terms of safeguarding patients’ right to informed consent, the application of new generation AI software such as DeepSeek will challenge doctors’ information disclosure rules, posing fundamental challenges to the accuracy, adequacy, and objectivity of doctors’ disclosure of information, while also affecting patients’ abilities and willingness to consent (30). In terms of liability for infringement, the definition of liability for medical AI infringement, algorithm transparency and legal disputes are new challenges. On the one hand, in the event of medical AI misdiagnosis, the responsibility delineation among doctors, hospitals, and AI software developers becomes more complex. On the other hand, when patients question their diagnosis and treatment behavior based on DeepSeek search results, it may also interfere with the normal process of determining medical liability, mislead the judgment of appraisers, and make the responsibility determination which should be based on professional medical knowledge and clinical practice become more complex. For example, patients may find that a certain medication should be the preferred treatment in specific situations according to the DeepSeek search results, but doctors may not choose it based on the patient’s actual condition. If the patient’s condition is not well controlled, how to eliminate these inappropriate interferences and accurately judge the rationality of the doctor’s diagnosis and treatment behavior becomes a new challenge in determining responsibility.

## 3 Discussion

This section will analyze the main medical damage liability risks of medical AI. Although people are concerned about the legal risks of deploying DeepSeek in hospitals, so far there have been no lawsuits. The cases in the fake news mentioned above can be used as a hypothetical case to discuss about the legal liability for medical AI misdiagnosis. It reflects the difficulties in defining the legal responsibilities of medical AI. The Civil Code of China adopts a broad definition of medical liability, including compensation for damages caused by violations of medical treatment obligations liability (diagnosis and treatment damage liability), compensation liability for breach of disclosure obligation (medical ethic liability), medical product liability and compensation liability for infringement of patients’ privacy rights and personal information damage. Below is a detailed discussion on these four types of medical liabilities.

### 3.1 Medical product liability

Is AI a ‘doctor’ or a ‘tool’? Is AI medical software a medical device? Although we often hear the expression of AI doctors, AI doctors are not traditional human doctors, but medical auxiliary diagnostic systems developed based on AI technology (31). So, does this auxiliary diagnostic system belong to medical equipment? Should AI companies and hospital doctors be held responsible for medical products liability when misdiagnosis and infringement occur?

#### 3.1.1 The legal nature of medical AI software

According to the “Guiding Principles for Classification and Definition of Artificial Intelligence Medical Software Products” issued by the National Medical Products Administration (“NMPA”) on July 1, 2021, AI medical software refers to independent software that uses AI technology to achieve its medical purposes based on medical device data.

“Medical device data” refers to objective data generated by medical devices for “medical purposes” according to the “Guidelines for Registration and Review of Artificial Intelligence Medical Devices” issued by the NMPA on March 7, 2022. Medical image data generated by medical imaging equipment (such as X-rays CT, MRI), physiological parameter data generated by medical electronic devices (such as electrocardiogram, blood pressure, etc.) are medical device data; In special circumstances, objective data generated by general equipment for medical purposes also belong to medical device data, such as skin photos taken by digital cameras for skin disease diagnosis, electrocardiogram data collected by health electronic products for heart disease warning, etc. (32). “Based on medical device data” includes the generation and use of medical device data, including the use of medical device data alone or the combination of medical device data with non-medical device data (such as patient complaint information, inspection report conclusions, electronic medical records, medical literature, etc.). “Medical purpose” usually include: diagnosis, prevention, monitoring, treatment or relief of diseases; Diagnosis, monitoring, treatment, alleviation, or functional compensation of injuries; The examination, substitution, regulation, or support of physiological structures or processes; Support or maintenance of life; Pregnancy control; And providing information for medical or diagnostic purposes by examining samples from the human body (33).

Does medical artificial intelligence software belong to medical devices? The current legal dilemma is how to view the “tool” attribute of generative AI medical software? Its self-learning ability blurs the boundary between “product” and “service.” As AI systems continue to update and iterate algorithms, the original training data and current decision logic are difficult to trace (34).

The Guiding Principles for Classification and Definition stipulates that medical AI software is managed as Class III and Class II medical devices based on whether it is used to assist decision-making. For AI medical software with low maturity in medical applications (referring to those that have not been marketed or whose safety and effectiveness have not been fully proven), if used to assist decision-making, such as providing clinical diagnosis and treatment recommendations such as lesion feature recognition, lesion nature determination, medication guidance, and treatment plan formulation, it shall be managed according to the Class III of medical devices; If used for nonauxiliary decision-making, such as providing clinical reference information for data processing and measurement, it shall be managed according to Class II of medical devices.

Therefore, if the processing object of a software product is medical device data, and it is used for medical purposes, it complies with the definition of medical devices in the RSAMD and is managed as a medical device. If the processing object of a software product is non-medical device data (such as patient complaints and other information, inspection report conclusions), or its core function is not to process, measure, model calculate, analyze medical device data, or not used for medical purposes, it will not be managed as a medical device. For example, hospital information management systems, diagnosis and treatment appointment systems, medical insurance payment or accounting systems will not be classified as medical devices if they do not meet the above requirements.

At present, hospitals in China have deployed DeepSeek on a large scale and applied it to decision support, assisted decision-making, and diagnosis and treatment. How to determine whether DeepSeek is a medical device? The analysis principles of the Clinical Decision Support Software (CDS) by the US Food and Drug Administration (FDA) provide reference for whether such artificial intelligence technology applications belong to medical devices (35). If the software provides supportive suggestions for medical professionals, rather than single, specific, selected outputs or instructions, healthcare personnel still need to make comprehensive judgments based on multiple factors, rather than relying solely on the conclusions given by the software, then it may not need to be regulated as a medical device. If the software allows healthcare professionals to independently review the basis of their recommendations, and healthcare professionals do not primarily rely on these recommendations in clinical decision-making, they may not need to be regulated as medical devices. The EU’s Medical Device Regulation (MDR) defines medical devices as instruments, devices, equipment, software, implants, reagent materials, and other items intended for use by humans. The intended use of these devices is determined by the manufacturer, whether used alone or in combination, for the purpose of diagnosing, testing, or treating diseases, injuries, or disabilities. So medical AI software that meets these purposes belongs to the category of medical devices and is naturally a product.

Overall, whether the DeepSeek software deployed in hospitals is recognized as a medical device depends on whether it processes medical data for medical purposes and whether medical professionals primarily rely on this data for clinical decision-making. From the current situation, unlike medical devices with built-in AI software, DeepSeek mainly provides information and advice, and the diagnosis and treatment results are mainly handled by doctors. Medical devices with built-in AI software that comply with the “Guiding Principles for Classification and Definition of Artificial Intelligence Medical Software Products” mentioned above need to apply for registration as medical devices. If Deepseek is built into a product for medical purposes to process medical data, and doctors’ diagnoses mainly rely on its data recommendations, then the product containing DeepSeek software should be included in medical device regulation.

#### 3.1.2 Medical AI software’s medical product responsibility

Medical product liability refers to the use of defective drugs, disinfectants, medical devices, and other medical products by medical institutions during the medical process, resulting in harm to patients. For example, using substandard medical equipment or drugs that cause harm to patients (36). In this case, medical institutions, medical product producers, or sellers are required to bear corresponding medical damage compensation responsibilities. The determination of product liability is mainly based on the definition of product liability stipulated in the Civil Code and the Product Quality Law of China. If the medical AI software is regulated as a type of medical product, and causes harm to others due to defects in the product, the infringed party can request compensation from the producer or seller of the AI software or the user (medical institution).

Unlike fault liability in Diagnosis and treatment damage liability, medical product liability is a strict liability. That is, regardless of whether the producer is at fault or not, as long as the product has defects that cause personal or property damage to others, the producer must bear the liability for compensation. This form of liability is mainly applicable in the field of product liability, especially in the case of damage caused by defective products. Therefore, whether to assume responsibility for medical products mainly depends on determining whether medical AI software is a defective product and the causal relationship between the consequences of damage and product defects.

How to determine product defects? The existing product defect judgment standards are “technical standards,” which are applicable to traditional performance stable products. However, facing to the characteristics of medical AI self-learning and updating, the “technical standards” faces application difficulties in medical AI product defect judgment. For the identification of defects in medical AI software for diagnosis and treatment, the “rational algorithm” standard should be adopted, which means that the performance of AI for diagnosis and treatment should meet the level that a rational algorithm should possess, otherwise there is an unreasonable danger. Deriving “rational algorithm” standard from “rational person” standard can effectively evaluate the “behavior” of algorithms directly to adapt to the intelligent attributes of diagnostic and therapeutic artificial intelligence. The “rational person” standard refers to the situation where a rational person could foresee the occurrence of harm, but the actor should have foreseen it but did not foresee it, or foreseen it but did not avoid the occurrence of harm, then the actor did not fulfill the duty of care. In fact, whether it is the rational person standard or the rational algorithm standard, they are essentially imposing general social expectations on the actor, but the object of the rational person standard is the actor, while the rational algorithm standard targets the autonomous algorithm ontology (37). But the algorithm itself is not human and cannot impose a duty of care on it. Only the reasonable expectations of society towards algorithms can be included in the evaluation of whether algorithms are reasonable. If the performance of AI in diagnosis and treatment is far inferior to that of medical personnel, such as frequent missed diagnosis of obvious lesions by the system, and the prescribed prescriptions often violate basic medical principles and cannot provide the necessary assistance, then it can be considered that it does not meet the standards of rational algorithms and poses unreasonable risks. Of course, this example is too simplistic, and medical AI that develops such algorithms in practical life should not be able to enter the market after evaluation.

How to determine a “rational algorithm” specifically? It is difficult to find suitable reference points for comparing with other similar algorithms, and it is also impossible to objectively evaluate complex algorithms when compared with human doctor behavior (as shown in the example above). The normative standard of “rationality” itself is also a vague concept, which changes with different legal values. A “rationality” may be the idea of equality and freedom, where individuals must act in a way that allows them to coexist freely with others. Therefore, a reasonable duty of care is the harmonization of human freedom and the protection of others from harm caused by free behavior (38). Another possibility is that rationality must achieve the maximization of economic efficiency (welfare). When preventive measures are taken to prevent damage at a cost lower than expected, compensation liability must be borne. In reality, the normative standard of “rationality” serves as an intermediary between legal norms and free evaluation of evidence, allowing judges to find a method guide when judging the behavior of parties. For example, when the court applies the standards of a rational person, it often judges how a rational person would behave under the same conditions and knowledge. Although rational standards are difficult to describe in legislation and have practical complexity, they are ultimately normative, abstract, and objective, while also considering the cognitive state of specific actors. The standard for “rational person” needs to be analyzed by combining individual case factors to determine what abilities and knowledge “rational person” possess. Ultimately, it is necessary to rely on the psychological mechanism of the judge to judge the rational person who has been concretized. Similarly, the judgment of “rational algorithms” also needs to be analyzed in specific cases to determine what abilities “rational algorithms” possess. The participation mechanism of algorithms is a prerequisite for algorithm accountability (39). The collaboration between algorithms and humans, that is, the participation of algorithms in decision-making, is currently the optimal mode in many works. The behavior and choices of algorithm participation in decision-making have a completely different structure from human internal collaboration. The requirements for the duty of care reflected in the reasonable algorithm standards vary in different human-machine cooperation modes.

It should be noted that AI systems themselves do not have legal subject qualifications, and if algorithms do not meet the corresponding rationality standards, they ultimately need to return to the responsibility of algorithm developers. The standard of “rational algorithm” is to avoid unreasonable dangers. The design program of the algorithm reflects the will of the developer. In the case where it is impossible to evaluate the performance and quality of an AI product, the rationality of the developer or producer should be considered, that is, whether the developer has fulfilled their duty of care to ensure the reasonable expectations of users and personal and property safety. In this way, the determination of product defects and causal relationships is focused on the duty of care of algorithm developers.

The duty of care for algorithm developers is whether they have complied with the regulatory requirements of relevant laws for medical AI. Specifically, medical AI algorithm developers should fulfill their duty of care in accordance with regulatory requirements at every stage, such as: in the design and development process, algorithm developers should fulfill their due diligence obligations in terms of algorithm data sources, algorithm model selection, algorithm decision fairness, etc., to ensure that the output results of diagnostic and therapeutic AI algorithms meet transparent, accurate, and fair standards; In the process of entering the market, the algorithm developers, producers, and sellers should fulfill their corresponding warning and explanation obligations to medical institutions and their medical personnel, so that medical personnel can clearly and fully understand the scope of application, usage methods, basic operational logic, and the basis for conclusions drawn from diagnostic and therapeutic artificial intelligence; In the process of subsequent usage, the developers, producers, and sellers should fulfill their obligation to track and observe artificial intelligence systems in a timely manner to ensure the safe and stable operation of diagnostic and therapeutic artificial intelligence systems. If the developer, producer, and seller fail to fulfill their corresponding level of duty of care, it can be determined that there is a product defect.

That is to say, the more detailed the regulatory requirements for medical AI, the clearer the judgment of medical product liability. In the process of large-scale deployment of DeepSeek, it is urgent to establish detailed regulatory guidelines for the use of DeepSeek by hospitals. We can draw some references from the MDCG 2025–6, namely Interplay between the Medical Devices Regulation (MDR) & *In vitro* Diagnostic Medical Devices Regulation (IVDR) and the Artificial Intelligence Act (AIA), which is endorsed by the Artificial Intelligence Board (AIB) and the Medical Device Coordination Group (MDCG), setting clear regulatory framework for Medical Device Artificial Intelligence (MDAI). The MDR and IVDR require manufacturers to manage the entire lifecycle of MDAI, ensuring that the MDAI remains safe and performant throughout its use. AIA specifically focuses on AI systems, emphasizing 13 aspects such as data governance, transparency, and human supervision. The MDAI manufacturers should integrate AIA requirements into existing MDR/IVDR quality management systems.

Medical product liability and diagnosis and treatment damage liability do not always occur independently, but rather interact and overlap in practice, leading to uncertainty in the attribution of responsibility for AI medical care (40). If AI developers have certain flaws in algorithm design and data processing, or AI systems have biases in training data, resulting in diagnostic accuracy for specific diseases not meeting industry standards, the developers need to bear part of the responsibility and provide corresponding compensation to patients. Medical institutions that did not conduct sufficient evaluation and validation when introducing this AI diagnostic system, and lacked effective regulatory mechanisms during its use or doctors who overly rely on AI results during the diagnosis process and fail to fully utilize their professional judgment abilities have also been identified as at fault. The medical institutions and doctors should jointly bear another part of the responsibility and compensate patients accordingly.

At present, the determination of legal responsibility should focus on the actual role of AI systems in the entire diagnostic process. If the AI system is only used as an auxiliary tool, doctors have a responsibility to carefully judge and verify the results they provide. If doctors fail to fulfill this responsibility, they will be judged to bear the main responsibility. AI developers and medical institutions, if they can prove that they have fulfilled their reasonable duty of care, will bear relatively lighter responsibilities.

### 3.2 Diagnosis and treatment damage liability

Diagnosis and treatment damage liability refers to the negligent behavior of medical behavior that does not conform to the medical professional knowledge and technical level at that time (41). For example, errors made by doctors during diagnosis and treatment, or improper use of medical equipment, can cause harm to patients. In this case, medical institutions need to bear corresponding liability for infringement compensation. The Civil Code stipulates that the focus of liability for medical damages lies in “fault,” that is if the patient suffers damage during the diagnosis and treatment activities and the medical institution or its medical staff are at fault, the medical institution shall bear the liability for compensation. Starting from the human-machine interaction relationship of AI healthcare in practice, faced with the assistance of DeepSeek in diagnosis and treatment, should medical personnel adhere to their own judgment or adopt machine judgment? How to determine the diagnosis and treatment negligence of medical personnel if their adoption or refusal of machine judgment causes harm to patients?

As mentioned above, AI assisted diagnosis technology is an auxiliary diagnosis and clinical decision support system, which cannot be used as the final clinical diagnosis, but only as a clinical auxiliary diagnosis and reference. The final diagnosis must be determined by qualified clinical physicians. AI is only an auxiliary tool to improve the efficiency of medical personnel’s judgment and display information in the medical process. In the case of using AI assisted programs for diagnosis and treatment, the main body implementing the diagnosis and treatment behavior is medical personnel, who need to bear the ultimate responsibility for their judgments.

The complexity and unknowns of diagnostic and therapeutic activities determine that machine judgment is not always true, and medical personnel should have the freedom to exercise discretion, including whether to use diagnostic and therapeutic AI and how to treat machine judgment. Adhering to the auxiliary positioning of AI in diagnosis and treatment is ultimately to ensure that machine judgments are safely transformed into diagnostic and treatment measures. Medical personnel need to fully respect the AI in diagnosis and treatment and bear appropriate re judgment obligations. If there is a disagreement between humans and machines, medical personnel have the final decision-making power, but they must fulfill the duty of care of a rational doctor. Fully respect the discretion of medical personnel, as long as the treatment plan chosen by medical personnel can receive a considerable number of recognized and respected colleagues’ support, even if there are other treatment plans and it is later proven that the medical personnel are wrong, their negligence cannot be considered (42).

The basic standard for determining diagnosis and treatment negligence should be the “reasonable doctor’s standard” (43). Article 1,221 of the Civil Code regards the standard of medical proficiency at that time as the basic criterion for determining diagnosis and treatment negligence. Article 16 of the “Interpretation of the Supreme People’s Court on Several Issues Concerning the Application of Law in the Trial of Medical Damage Liability Disputes” clearly stipulates that in addition to applying the medical level standards at that time, the judgment of medical staff’s diagnosis and treatment negligence also needs to be based on laws, administrative regulations, rules, and other relevant diagnosis and treatment norms, while comprehensively considering factors such as the urgency of the patient’s condition, individual differences among patients, local medical level, medical institutions, and medical staff qualifications. The above provisions can infer that the basic standard for diagnosis and treatment of negligence is the “reasonable doctor standard,” which examines whether medical personnel have fulfilled the duty of care that a rational doctor should have in the same or similar circumstances in a specific case, and have demonstrated the appropriate level of diagnosis and treatment. If medical personnel reasonably adopt new therapies based on comprehensive comparison, even if they cause damage, they should not be deemed to have diagnostic and therapeutic negligence. On the contrary, if medical personnel ignore the new treatments provided by machine judgment and stick to traditional treatment plans in pursuit of absolute safety, they may be deemed to have diagnostic and therapeutic errors.

In the hypothetical case, the “Intelligent Diagnosis AI System” company argues that the system has labeled the “auxiliary tool” attribute and the training data covers millions of cases, and “misdiagnosis is a statistical probability problem,” so it is not at fault. According to Article 1,203 of the Civil Code, if the plaintiff cannot prove the existence of design defects in the AI system, the responsibility shall be borne by the user. The hospital believes that the AI diagnostic process complies with the “Regulations on the Management of Artificial Intelligence Applications in Medical Institutions (Trial),” and doctors have the final decision-making power. But the court found during questioning that 80% of the hospital’s initial cases rely on AI generated reports, and the average review time for doctors is less than 2 min. If doctors fail to conduct necessary reviews of AI recommendations, it may constitute a “failure to exercise due diligence” as stipulated in Article 24 of the Physician Law.

In the context of localizing the deployment of DeepSeek in hospitals, although there have been no judicial disputes related to medical diagnosis so far. However, if an individual uses DeepSeek for medical purposes, DeepSeek is not responsible because it has clearly informed the source party from the beginning for reference only and cannot be used as a direct diagnosis and treatment plan. DeepSeek does not have prescription authority, and its advice is used as a medical recommendation. Without the approval of a professional doctor, it cannot be used as a professional diagnostic plan. Doctors need to follow the “reasonable doctor standards” and be responsible for adopting or not adopting DeepSeek’s diagnosis and treatment recommendations.

Unlike medical product liability, medical diagnostic liability focuses on the determination of the negligence causing damages by medical institutions and personnel. For doctors, excessive reliance on AI diagnosis or unverified system results (such as not re-examining suspicious nodules) may be considered as diagnostic and therapeutic negligence. For medical institutions, when hospitals apply diagnostic and therapeutic artificial intelligence, their organizational management obligations are correspondingly strengthened. If a medical institution fails to provide training and management for medical personnel, or fails to update and maintain the system in a timely manner, resulting in patient damage, according to Article 1,218 of the Civil Code, the medical institution shall be liable for compensation. If medical personnel have intentional or gross negligence, such as failing to fulfill their obligation to re-evaluate suggestions that clearly violate medical knowledge provided by the system, resulting in patient damage, medical institutions may seek compensation from the medical personnel in accordance with Article 1,191 of the Civil Code after assuming liability for compensation.

### 3.3 Medical ethics liability

Medical ethics liability involves medical personnel not fully informing patients of their condition, not providing timely and effective medical advice, or not keeping confidential information related to their condition during medical behavior. For example, doctors who fail to inform patients of surgical risks or perform surgeries without obtaining their consent violate medical professional ethics and conscience. In this case, medical institutions also need to bear the liability for infringement compensation.

Medical AI poses fundamental challenges to the accuracy, adequacy, and objectivity of information disclosed by doctors, while also affecting patients’ abilities and willingness to consent (44). Article 33 of the “Regulations on the Administration of Medical Institutions” (45) requires medical institutions to respect patients’ right to know about their symptoms, diagnosis, and treatment. When performing surgery, special examinations, or special treatments, patients’ consent must be obtained; When it is impossible to obtain the patient’s opinion, the consent and signature of the family or related parties should be obtained. In the promotion of medical AI, patients can only make rational decisions with full knowledge. Ensuring informed consent of patients in the field of medical artificial intelligence can ensure that their rights are fully protected. Article 13 of the “Regulations on the Prevention and Handling of Medical Disputes” (46) stipulates that medical personnel shall explain the condition and medical measures to patients during diagnosis and treatment activities. If special examinations or treatments that pose certain risks and may have adverse consequences, such as surgery or clinical trials, need to be performed, medical personnel should promptly explain the medical risks, alternative medical plans, and other situations to patients and obtain their written consent.

In a medical damage liability dispute case (47) filed by a patient against a hospital, the court held that the hospital had not fulfilled its obligation to inform about medical risks before surgery. According to Article 55 of the Tort Liability Law, medical personnel shall explain the condition and medical measures to patients during diagnosis and treatment activities. If surgery, special examination, or special treatment is required, medical personnel shall promptly explain the medical risks, alternative medical plans, and other situations to patients and obtain their written consent; If it is not appropriate to explain to the patient, it should be explained to the patient’s close relatives and their written consent should be obtained. If medical personnel fail to fulfill their obligations under the preceding paragraph and cause harm to patients, medical institutions shall bear the liability for compensation. In this case, the hospital needs to use a stereotactic machine system (a surgical robot) to insert electrodes into the patient’s skull, which is an invasive examination. There is a certain medical risk to whether the patient’s preoperative condition can be located for intracranial lesions using the stereotactic machine system. The hospital should promptly explain the risk to the patient’s guardian to obtain their valid consent. The hospital did not inform the patient’s guardian in the surgical signature document for electrode placement that the stereotactic machine system would be used to locate intracranial lesions for the patient, which violated their right to know. The above actions of the hospital deprived the patient and their close relatives of the right to choose treatment plans based on medical risks, and thus lost the opportunity to choose other treatment methods that may result in lower trauma. In addition, the hospital claimed to be a tertiary hospital when promoting to the public, but in reality, the hospital is only an ordinary hospital and has not yet determined its level. The hospital’s exaggerated promotion has affected the correct assessment of medical risks by patients and their families, causing them to lose the opportunity and right to choose other treatment hospitals that may result in lower trauma.

Unlike the use of diagnostic robots, after the large-scale deployment of DeepSeek in hospitals, the situation of doctor-patient communication has changed. On the one hand, the information disclosed by doctors is easily questioned for lacking accuracy and efficiency. It has become easy for patients to obtain massive medical information through AI retrieval tools. By entering keywords such as symptoms, signs, and disease names, patients can quickly obtain a large amount of relevant medical information, including disease diagnosis criteria, treatment methods, medication use, surgical plans, and more. Patients are no longer passively listening to doctors’ advice, but more actively using the information they obtain to communicate with doctors. The number of patients questioning doctors’ diagnosis and treatment behavior based on AI search results is increasing, and there are even cases where patients upload the original medical records to DeepSeek to “judge” whether the medical institution is at fault. For example, a husband of a cancer patient approached a doctor with the results of DeepSeek, believing that the examination given to his wife by the doctor was an excessive examination. If patients find that doctors have not mentioned certain treatment methods or information due to DeepSeek search results, they may claim that doctors have violated their right to know. However, doctors follow professional diagnosis and treatment standards and clinical experience during the diagnosis and treatment process, and may not actively mention some treatment methods or information that are not suitable for the patient’s individual situation or even have obvious contraindications. This has led to controversy over whether doctors have violated patients’ right to know. The lack of clear standards in the law to define the boundary of doctors’ disclosure obligations and the impact of DeepSeek retrieval information poses difficulties for judicial practice.

On the other hand, how can doctors ensure that the information disclosed is accurate and sufficient when using DeepSeek’s answers to provide diagnostic advice to patients? The complexity of medical AI weakens the ability of patients to provide informed consent. Medicine itself is characterized by theories, associations, and opaque decision-making processes, coupled with the complexity of medical AI systems, making it more difficult for patients to understand medical procedures (48). For practitioners of medical ethics, the “black box” nature of AI and its obscure internal logic make it more challenging to explain necessary information to patients (49). Medical AI is a relatively new field, and many aspects have not been fully understood and validated. The opacity or un-explainability of AI systems further complicates patients’ access to necessary information to make medical decisions, thereby weakening their decision-making autonomy.

In the context of medical artificial intelligence, it is imperative to reconstruct patient consent rules. Firstly, improve relevant legal provisions. On the one hand, clarify the evidential value of AI applications in medical disputes. It should be clarified that the AI information searched by patients can only be used as auxiliary reference and cannot be directly used as a basis for determining medical behavior errors. On the other hand, it is necessary to refine the specific content and standards of doctors’ disclosure obligations, clarify under what circumstances doctors must disclose specific medical information to patients, and balance routine disclosure with special disclosure obligations caused by individual differences, in order to reduce legal disputes arising from the right to know.

Secondly, pay attention to patient science popularization and information guidance. Medical institutions and healthcare professionals should proactively assist patients in correctly understanding the limitations of AI retrieval of information. During the outpatient or inpatient process, use promotional brochures, video playback, and other forms to educate patients on how to screen reliable sources of medical information, as well as the reasons why AI search results cannot replace professional doctors’ diagnosis, guiding patients to rationally view AI retrieval information, and establish correct medical concepts.

Thirdly, strengthen the training of medical institutions and medical personnel. Medical institutions should strengthen the training of medical personnel and improve their ability to respond to patients’ AI based retrieval and questioning. The training content should include: (1) legal knowledge to enable medical personnel to understand the legal risks and responsibilities when facing such situations; (2) Communication skills training, enabling medical staff to explain professional medical issues to patients in a simple and understandable way, resolving patient doubts, such as using cases, metaphors, and other methods to explain the necessity of personalized treatment to patients; (3) Strengthen the updating of professional knowledge to ensure that medical personnel can respond to patient inquiries with reason and evidence based on the latest medical developments and clinical practices, and safeguard their legitimate rights and interests.

In general, under the patient-centered standard, doctors should communicate effectively in a targeted manner based on individual factors such as the patient’s physical and mental health status, personal preferences, and understanding ability, while ensuring the adequacy of disclosed information. Matters that can bring significant risks or benefits to patients are classified as’ substantive information ‘and should be transparently communicated. This includes not only current medical practice information, but also the details of the use of medical AI in assisted diagnosis. The medical institution should actively participate in the formulation of relevant specifications for medical information on the Internet, improve the quality and accuracy of medical information retrieved by AI, and reduce the doctor-patient conflicts caused by misleading information from the source.

### 3.4 Data compliance responsibility of medical AI

In addition to the responsibility determination issues mentioned above, data compliance of AI medical is also a legal risk that needs to be taken seriously. If the use of medical AI violates patients’ privacy rights and personal information, corresponding compensation responsibilities should also be borne. There are certain differences between DeepSeek’s localized deployment model in hospitals and medical devices such as auxiliary diagnosis and treatment systems and intelligent decision-making platforms under the Regulations on the Supervision and Administration of Medical Devices. The current regulatory framework also lacks targeted regulatory guidance for such innovative forms.

Generally speaking, the localization deployment of DeepSeek in hospitals does not involve data “discharge,” but rather trains locally deployed AI programs based on the hospital’s own past case database (50). According to the Personal Information Protection Law of China and the Guidelines for Health and Medical Data Security of Information Security Technologies, various types of information related to identified or identifiable natural persons that have not been anonymized and recorded in electronic or other ways are considered personal information. Personal information related to medical and health care is considered sensitive personal information, and the processing of sensitive personal information must comply with requirements such as “obtaining individual consent” and “informing of the necessity of processing sensitive personal information and its impact on personal rights and interests.” In addition, the new “Regulations on the Management of Medical Records in Medical Institutions” stipulates that medical institutions and their medical staff should strictly protect patient privacy and prohibit the disclosure of patient medical records for non-medical, teaching, and research purposes.

In the process of localizing DeepSeek in hospitals, if it is required to register/file medical devices under the current regulatory framework, it will face practical obstacles such as long verification cycles and difficulties in registration and application; But if the regulatory control over medical AI technology is detached from the current regulatory framework, the inherent risks of AI illusions and other AI technologies may lead to an increase in the probability of medical decision-making errors. In the process of using past cases to perform incremental AI training, the relevant provisions of the law and regulations should be followed first to avoid leaking patient medical record data and avoiding data “discharge.” Hospitals may consider desensitizing, cleaning, and annotating case data before generating training samples. For the secondary utilization of health and medical data (with different purposes from when the data was collected), corresponding de-identification work should be carried out on the data. The de-identification rules should meet the principle of minimum count, such as no less than 5 people meeting the same description after de identification (51).

## 4 Conclusion

AI represented by DeepSeek has significant reference value in the medical field that cannot be ignored. In terms of disease diagnosis, it can quickly analyze massive case data, explore potential disease patterns, help doctors understand the condition more comprehensively, and assist in the diagnosis of rare or complex diseases. In the formulation of treatment plans, AI can integrate the latest medical research results from around the world, providing doctors with more treatment ideas and reference plans. For example, in tumor treatment, AI can recommend personalized treatment plans based on patients’ genetic data, disease development stages, and other information.

Although AI can provide reference, in actual medical decision-making, the professional judgment and responsibility of doctors cannot be replaced. Medical AI is not about “doctors being replaced” (52), but about “doctors being liberated.” It is not intended to replace top doctors, but rather targets a large number of “common diseases” scenarios in primary healthcare, especially in areas where there is a shortage of doctors, weak diagnostic and treatment capabilities, and insufficient information technology, realizing sustainable healthcare (53). Future breakthroughs should focus on the “triple defense line” of technology, law, and insurance. At the technical level, establish an “algorithm audit” mechanism, such as annual review of high-risk algorithms by the National Medical AI Ethics Committee, with a focus on evaluating indicators such as bias rate and interpretability. At the legal level, introducing the principle of “presumption of fault” and strengthen data compliance and privacy protection. At the insurance level, “AI medical liability insurance” can also be developed, with premiums dynamically adjusted based on algorithm transparency and misdiagnosis history.

“We urgently need to establish a ‘human-machine co-governance’ framework: AI is responsible for probability, while humans are responsible for causality; AI provides options, humans make choices.” Medical AI is not only a dispute over the division of legal responsibilities, but also a reexamination of human technological rationality. We should reshape the development trajectory of medical AI - finding the balance between efficiency and safety, innovation and responsibility that cannot be lost.

## Data Availability

Publicly available datasets were analyzed in this study. This data can be found at: https://flk.npc.gov.cn/index.
